# Determinants of Helminthic Infections and Anemia among Schoolchildren in Bahir Dar Zuria District, Northwest Ethiopia

**DOI:** 10.1155/2021/9913118

**Published:** 2021-09-29

**Authors:** Yeshimebet Fetene, Tadesse Hailu, Mulat Yimer, Megbaru Alemu

**Affiliations:** ^1^Amhara Public Health Institute, Bahir Dar City, Ethiopia; ^2^Department of Medical Laboratory Science, College of Medicine and Health Sciences, Bahir Dar University, Ethiopia

## Abstract

**Background:**

Soil-transmitted helminths and *Schistosoma mansoni* are the major helminthic parasites that cause major public health problems among schoolchildren in developing countries. Infection with the above parasites decreases the hemoglobin level of children. However, information regarding the current status of helminthic infections and anemia is limited. Hence, this study aimed to assess the prevalence and determinants of helminthic infections and anemia among children.

**Methods:**

A cross-sectional study was conducted among 394 schoolchildren at Sekelet primary school in northwest Ethiopia, from February to March 2017. Study participants were selected by a systematic random sampling technique. Stool samples were collected and processed via the modified Ritchie's concentration technique to detect parasites in stool. A HemoCue Hb 201 analyzer was used to determine the hemoglobin level. Data were analyzed using SPSS version 23 statistical software. Association of helminthic infections and anemia with independent variables was determined using logistic regression analysis. Variables with *P* < 0.05 were considered statistically significant.

**Results:**

From the total of 394 participants, 185 (46.9%), 164 (41.6%), and 112 (28.4%) were infected with intestinal parasites, helminths, and STHs, respectively. The prevalence of hookworm and *Schistosoma mansoni* were 106 (25.6%) and 54 (13.7%), respectively. The prevalence of anemia among the schoolchildren was 278 (70.6%). Anemia was prevalent among 55 (51.9%) hookworm-infected and 19 (35.2%) *S. mansoni*-infected children. Not wearing shoes and improper utilization of latrine were significantly associated (*P* < 0.05) with hookworm infection, and frequent swimming in the river was also significantly associated (*P* ≤ 0.001) with *Schistosoma mansoni* infection. *Schistosoma mansoni* and hookworm infections were also significantly associated (*P* ≤ 0.001) with low levels of hemoglobin.

**Conclusion:**

Hookworm and *Schistosoma mansoni* infections and anemia are highly prevalent among schoolchildren. Hookworm and *Schistosoma mansoni* infections are significantly associated with anemia. Therefore, helminthic detection and hemoglobin determination should be done simultaneously among schoolchildren.

## 1. Introduction

Helminthic infections, mainly soil-transmitted helminths (STHs) and *S. mansoni* are major public health problems in the world, especially in sub-Saharan Africa [1]. Globally, more than 1.5 billion people are infected with STHs, and over 267 million preschool-age children (PSC) and over 568 million school-age children (SAC) live in areas where the above parasites are intensively transmitted [2]. The number of people living in helminth endemic areas in Ethiopia was estimated to be 79 million, of which, PSC, SAC, and adults comprise the respective 9.1 million, 25.3 million, and 44.6 million [3]. Ethiopia harbors the second highest burden of ascariasis and stands third with the burden of hookworm infection, and 1/8th of the Ethiopian population is believed to be infected with *T. trichiura* [4].

The prevalence and distribution of helminthic infections varies from region to region due to several environmental, social, and geographical factors in Ethiopia [5]. Transmission of STHs is convened through a fecooral route (*A. lumbricoides* and *T. trichiura*) and through skin penetration (hookworms) [6]. Schistosomiasis is also transmitted by skin penetration of the cercaria during contact with freshwater [7]. Helminthic infections have immense negative impact in children as it can cause anemia, stunting, protein-calorie malnutrition, and poor cognitive development, as well as school absenteeism [8–10].

Anemia is one of the most common disease manifestations observed in the tropics. Several factors including hookworm and *S. mansoni* infections cause anemia especially among children [11]. This information indicates that the effects of hookworm and *S. mansoni* infections have a considerable impact on children's health. Children who have anemia and other parasitic infections are becoming stunted and underweight compared to those who do not have [12]. Anemia in infants and children is associated with increased mortality [13], growth retardation [12], delayed motor development, poor cognitive abilities, reduced school performance [14], and impaired immune response [15]. Although helminthic infection has a considerable effect on hemoglobin level, information regarding the association of helminthic infection with anemia is limited. According to the health institution information in the study area, the prevalence of hookworm and *S. mansoni* infections is high in the study area. These parasites among children significantly decrease the level of hemoglobin. However, information regarding the distribution of hookworm and *S. mansoni* infections and anemic status is limited in the study area. Hence, this study aimed to assess the prevalence and determinants of helminthic infections and anemia among schoolchildren in northwest Ethiopia.

## 2. Materials and Methods

### 2.1. Study Design, Area, and Period

A school-based cross-sectional study was conducted at Sekelet primary school, Bahir Dar Zuria district, northwest Ethiopia, from February to March 2017. Sekelet Kebele is located at the border of Lake Tana, and it is 41 km away from Bahir Dar City. The annual temperature ranges from 10 to 32°C, and the average annual rainfall is 820-1250 mm. There is only one primary school and health center in Sekelet Kebele which is surrounded by Lake Tana.

The sample size was determined by a single population proportion formula (*n* = *Z*^2^*P*((1 − *P*)/*d*2)) using a previous (37%) helminthic infection prevalence [16]. A total of 394 apparently healthy schoolchildren were included in the study. All schoolchildren (6–14 years) who were included in the sampling process were actively learning during time of the data collection, and their parents volunteered to participate in the study. All schoolchildren who had taken anthelminthic drugs within the last three months prior to data collection time were excluded from the study. Among 15 classes in the school, 10 classes were selected by a simple random sampling technique. All the students in the selected classes were listed, and the participants were selected by finding the *K*-value. The students were proportionaly allocated in the selected classes based on the number of students in each class. The number of study participants in each selected class was selected by systematic random sampling techniques by taking the class roster as the sampling frame.

### 2.2. Data Collection

#### 2.2.1. Questionnaire

Data on the sociodemographic characteristics of study participants (age, sex, address, and religion) and associated factors of helminth infection were collected using a pretested structured questionnaire from the parents or guardians of the children. Trained nurses participated in the data collection.

### 2.3. Laboratory Data Collection

Freshly passed stool specimens were collected using clean plastic cups, and processed for microscopic examination. Due to limited budget and resources, a single stool sample was collected and a single slide per stool was prepared using the modified Ritchie concentration method.

#### 2.3.1. Modified Ritchie's Concentration Method

Approximately 1 ml of ethyl acetate and 2.5 ml of formalin were mixed in the sample collection tube. Then, half a gram of stool was mixed with the acetate-formalin solution in the sample collection tube and left to stand for a minute. Both pieces of the device were screwed and centrifuged at 1000 rpm for 3 minutes. The sample collection tube and filtration concentration unit were discarded. The supernatant was removed and a small amount of sediment was put on a microscopic slide. Detection of parasites in stool was done by using a microscope with 10x and 40x objectives.

#### 2.3.2. Hemoglobin (Hgb) Determination

Hgb level was determined using a portable hemoglobin spectrophotometer (the HemoCue Hb 201 Analyzer (HemoCue, Angelholm, Sweden) and a specially designed microcuvette (the HemoCue Hb 201 Microcuvette, HemoCue, Angelholm, Sweden). Then, anemia was defined as Hgb level less than 11 g/dl [17].

### 2.4. Data Quality Assurance

Training was given for data collector nurses and laboratory professionals. Pretest was done prior to data collection. The quality of reagents and instruments was checked by following standard operating procedures (SOP). Specimens were checked for labeling, serial numbers, and the quantity of stool samples.

### 2.5. Data Analysis

Data entry, coding, and cleanings were done and data processing was also done using EPI INFO version 6 and exported to SPSS version 23.0 software packages for statistical analysis. The magnitude of the helminth infection and anemia was summarized via descriptive statistics. Association of helminth infections and anemia with associated factors was determined by univariable logistic regression. Dependent variables with *P* < 0.2 in the univariable logistic regression were taken to multivariable regression analysis to control potential confounders at 95% CI. *P* values less than 0.05 were taken as statistically significant.

### 2.6. Ethical Consideration

Ethical clearance was obtained from the Ethical Review Committee of the College of Medicine and Health Sciences, Bahir Dar University. A support letter was also secured from the Amhara Public Health Institute. Written informed consent was obtained from the parents/guardians after explaining the purpose and objective of the study. Confidentiality of the study participants about the results was kept. Students positive for helminthic infections were treated accordingly, and severely anemic children were referred to the Sekelet health center for further management.

## 3. Results

### 3.1. Sociodemographic Characteristics

A total of 394 school children participated in this study, of which 201 (51%) were females. The age of study participants ranged from 6 to 14 years with a mean of 11.32 years. Three hundred (76.1%) and 94 (23.9%) were found in the respective 10–14 and 6–9-year age groups. Majority of the participants (391 (99.2%)) were Orthodox Christianity followers, and all participants were rural dwellers.

### 3.2. Prevalence of Helminthic Infections

The overall prevalence of intestinal parasites and helminthic and STH infections was 185 (46.9%), 164 (41.6%), and 112 (28.4%), respectively. The prevalence of hookworm, *S. mansoni*, *E. histolytica/dispar*, *A. lumbricoides*, *H. nana*, and *G. lamblia* was 106 (26.9%), 54 (13.7%), 19 (4.8%), 6 (1.5%), 3 (0.8%), and 2 (0.5%), respectively. Single- and double-parasite infection prevalence accounted for 180 (45.7%) and 5 (1.3%) among students, respectively (Table 1). Majority of the participants 227 (57.6%) and 373 (94.7%) did not have the habit of wearing shoes but have the habit of swimming in bodies of water, respectively.

### 3.3. Prevalence of Anemia

The mean Hgb level of study participants was 12.34 g/dl (ranging from 8 to 17 g/dl). The prevalence of anemia was 278 (70.6%) among the study participants. Among the anemic schoolchildren, the prevalence of mild and moderate anemia was 268 (96.4%) and 10 (3.6%), respectively. The prevalence of anemia among hookworm- and *S. mansoni*-infected children was 55/106 (51.9%) and 19/54 (35.2%), respectively. The prevalence of anemia among helminth-, single-parasite-, and double-parasite-infected schoolchildren was 79/164 (48.2%), 90/180 (50.0%), and 4/5 (80.0%), respectively. All *A. lumbricoides*-infected schoolchildren showed 100% mild infections (Table 2).

### 3.4. Factors Associated with Hookworm Infection

In multivariate analysis, the odds of children participating in irrigation activity had 10.83 times (AOR = 10.83; 95% CI: 1.03-113.85) higher risk for hookworm infection than their counterparts. Children who did not wear shoes were also 5.62 times (AOR = 5.62; 95% CI: 3.03-10.43) more exposed to hookworm infection than children who wore shoes regularly. Similarly, schoolchildren who did not properly utilize toilets for defecation were 5.34 times (AOR = 5.34; 95% CI: 2.91-9.81) more at risk to acquire hookworm infection than those who properly utilized toilets (Table 3).

### 3.5. Factors Associated with Schistosomiasis

In both univariate and multivariate regression analyses, swimming habit was significantly associated with *S. mansoni* infection among students (*P* < 0.05). In multivariate regression, schoolchildren who practice swimming increased the odds of *S. mansoni* infection by 4.96 times (AOR = 4.96; 95% CI: 1.64-8.99) more than those who had not practiced swimming. The odds of schistosomiasis was 3.84 times (AOR = 3.84; 95% CI: 1.89-13.04) higher among schoolchildren who washed their clothes using surface water than their counterparts (Table 4).

### 3.6. Factors Associated with Anemia

Using univariate logistic regression analysis, *S. mansoni* and hookworm infections were significantly associated with anemia among the schoolchildren (*P* < 0.05) (Table 5). In multivariate analysis, schoolchildren who had *S. mansoni* infection were 4.48 (AOR = 4.48; 95% CI: 2.21-9.06) times more likely to develop anemia than their counterparts. Schoolchildren who had hookworm infection increased the odds of anemia 4.39 times (AOR = 4.39; 95% CI: 1.68-11.46) higher than those who did not have hookworm infection (Table 5).

## 4. Discussion

Helminthic parasites, especially STHs and *Schistosoma*, are the major parasites that cause disease in humans. In the present study, the prevalence of helminthic infection was 41.6%, which is lower than previous results of 67.9% in southern Ethiopia [18]. The variation might be due to differences in the implementation of water sanitation and hygiene (WASH) programs in different geographical locations.

The prevalence of hookworm in this study was 26.9%, which is lower than previous findings in Gorgora [19], Mirab Abaya [20], and Zarima town [21]. The observed differences in the rate of infection might be due to variations in soil types, land surface temperatures, atmospheric moisture, habit of shoe wearing, and implementation of WASH in different geographical settings.

In this study, the prevalence of *S. mansoni* was 13.7% among schoolchildren. This result is lower than findings from different parts of Ethiopia, such as northwest Ethiopia [22] and Wondo genet Zuria [23]. The lower prevalence in the present study might be due to the difference in the sanitation and hygiene practices conducted by the Amhara Regional Health Bureau.

The prevalence of anemia among schoolchildren was 70.6%. This result is higher than previous reports of anemia (41.4%) in northwest Ethiopia [11], in southwest Ethiopia (37.6%) [24], in Kinshasa (41.6%) [10], in Yemen (46.0%) [25], and in Egypt (59.3%) [26]. The difference might be due to the differences in dietary iron intake and nutrition, since all the participants were taken from the rural area where the possibility of malnutrition among schoolchildren is high.

In multivariable analysis, the odds of hookworm infection were 2.47 times higher among children who do not wear shoes regularly. This was in line with other studies [27, 28]. This might be because the filariform larvae of hookworms that reside in the soil can readily penetrate barefooted children.

In the present study, improper utilization of latrines was significantly associated (*P* ≤ 0.002) with hookworm infection. This is because open defecation can increase the incidence of hookworm infection in a certain population for two probable accounts. First, open defecation results in widespread soil contamination with hookworm infective forms (filariform larvae), which augments the chance of infection of individuals. Secondly, the longer the time of contact between human feet and contaminated soil (as seen during defecation), the higher the chance of the percutaneous penetration by the filariform larvae.

In the present study, swimming (*P* ≤ 0.002) and washing clothes using surface water (*P* ≤ 0.001) were significantly associated with *S. mansoni* infection. This report is supported by previous studies [21, 29–31]. The habit of frequent contact with cercariae-infested water during swimming and washing in river water might predispose children to *S. mansoni* infection.

In the present study, hookworm and *S. mansoni* infections were significantly associated (*P* ≤ 0.001) with anemia. This finding is consistent with previous reports [11, 32]. Since these two parasites consume human blood and disturb intestinal absorption, and since hookworm infection also causes intestinal bleeding, they might cause anemia especially among children.

## 5. Conclusion

The prevalence of hookworm and *S. mansoni* parasites and mild anemia are high among schoolchildren. Improper utilization of latrines and not wearing shoes are associated with hookworm infection. Swimming and washing clothes in surface water are also associated with *S. mansoni* infection. Hookworm and *S. mansoni* infections are also significantly associated with anemia. Therefore, detection of helminthic infection and hemoglobin determination should be done simultaneously to minimize the risk of anemia in children.

## Figures and Tables

**Table 1:** Distribution of intestinal parasites and *S. mansoni* infections in Sekelet Kebele primary schoolchildren in northwest Ethiopia.

Parasite species		Frequency *N* (%)
Helminths	Hookworm spp.	106 (26.9)
	*S. mansoni*	54 (13.7)
	*A. lumbricoides*	6 (1.5)
	*H. nana*	3 (0.8)

Protozoans	*G. lamblia*	2 (0.5)
	*E. histolytica*	19 (4.8)

Single infection		180 (45.7)

Double infection	Hookworm + *S. mansoni*	5 (1.3)

**Table 2:** The distribution of anemia across helminthic infections in Sekelet primary schoolchildren, northwest Ethiopia.

Parasitic spp.	Total pos (*N*, %)	Anemia status (*N*, %)			
		Normal (*N*, %)	Mild (*N*, %)	Moderate (*N*, %)	Total anemia (*N*, %)
Helminths	164 (41.6)	85 (21.6)	69 (17.5)	10 (2.5)	79 (20.0)
*Lumbricoides*	6 (1.5)	0	6 (1.5)	0	6 (1.5)
Hookworm spp.	106 (26.9)	51 (12.9)	45 (11.4)	10 (2.6)	55 (14.0)
*S. mansoni*	54 (13.7)	35 (8.9)	16 (4.0)	3 (0.8)	19 (4.8)
Single infections	180 (45.7)	90 (22.8)	83 (21.1)	7 (1.8)	90 (22.9)
Double infections	5 (1.3)	1 (0.3)	1 (0.3)	3 (0.7)	4 (1.0)
Total		116 (29.4)	268 (68.0)	10 (2.6)	278 (70.6)

**Table 3:** Univariate and multivariate analyses of factors associated with hookworm infection among Sekelet primary schoolchildren, northwest Ethiopia.

Variables	Response	Hookworm		COR (95% CI)	*P* value	AOR (95% CI)	*P* value
		Yes	No				
Age groups	6-9	29	65	1			
	10-14	77	223	0.77 (0.47-1.29)	0.323		

Sex	Male	57	144	1.16 (0.75-1.82)	0.507		
	Female	49	144	1			

Participate in irrigation	No	4	1				0.047
	Yes	102	287	11.24 (1.24-101.87	0.031	10.83 (1.03-113.85)	

Drinking water source	Tap	49	197	1			≤0.001
	Surface	57	91	2.52 (1.60-3.97)	<0.001	2.22 (1.33-3.70)	

Shoes wearing	Yes	90	151	1			≤0.001
	No	16	137	5.10 (2.86-9.11)	≤0.001	5.62 (3.03-10.43)	

Proper toilet utilization	Yes	17	145	5.31 (3.01-9.37)	<0.001	5.34 (2.91-9.81)	<0.001
	No	89	143	1			

**Table 4:** Univariate and multivariate analyses of factors associated with *S. mansoni* infection at the Sekelet primary school, northwest Ethiopia.

Variable	Response	*S. mansoni*		COR (95% CI)	*P* value	AOR (95% CI)	*P* value
		Yes	No				
Age	6-9	14	80	1.14 (0.59-2.20)	0.701		
	10-14	40	260				

Sex	Male	26	175	0.88 (0.49-1.56)	0.650		
	Female	28	165	1			

Source of drinking water	Tap	38	244	1		—	
	Surface	16	96	1.07 (0.54-1.89)	0.833		0.979

Water for washing cloths	Tap	12	18	0.20 (0.09-0.44)	≤0.001	3.84 (1.89-13.04)	≤0.001
	Surface	42	322	0.206 (0.093,0.457)			

Irrigation practice	Yes	53	336	0.63 (0.07-5.75)	0.683	—	0.711
	No	1	4	1			

Swimming	Yes	10	11	6.80 (2.73-16.93)	≤0.001	4.96 (1.64-8.99)	0.002
	No	44	329	1		1	

**Table 5:** Univariate and multivariate analyses of factors associated with anemia among Sekelet primary school children, northwest Ethiopia.

Variable	Responses	Hemoglobin		COR (95% CI)	*P* value	AOR (95% CI)	*P* value
		≤11 g/dl	>11 g/dl				
Age	6-9	64	30	0.90 (0.55-1.48)	0.68		
	10-14	211	89	1			

Sex	Male	139	62	0.94 (0.61-1.45)	0.78		
	Female	136	57	1			

*S. mansoni* infection	Yes	49	5	3.42 (1.32-8.86)	0.011	4.48 (2.21-9.06)	≤0.001
	No	252	88	1			

Hookworm infection	Yes	96	10	3.89 (1.93-7.82)	≤0.001	4.39 (1.68-11.46)	≤0.001
	No	205	83	1			

## Data Availability

The data is freely available from PubMed (https://www.elsevier.com/) and Bahir Dar University (https://bdu.edu.et/node/74).
